# Effects of Patchwise Sampling Strategy to Three-Dimensional Convolutional Neural Network-Based Alzheimer’s Disease Classification

**DOI:** 10.3390/brainsci13020254

**Published:** 2023-02-02

**Authors:** Xiaoqi Shen, Lan Lin, Xinze Xu, Shuicai Wu

**Affiliations:** Department of Biomedical Engineering, Faculty of Environment and Life Sciences, Beijing University of Technology, Beijing 100124, China

**Keywords:** neuroimaging, Alzheimer’s Disease, image patch, deep learning, convolutional neural networks

## Abstract

In recent years, the rapid development of artificial intelligence has promoted the widespread application of convolutional neural networks (CNNs) in neuroimaging analysis. Although three-dimensional (3D) CNNs can utilize the spatial information in 3D volumes, there are still some challenges related to high-dimensional features and potential overfitting issues. To overcome these problems, patch-based CNNs have been used, which are beneficial for model generalization. However, it is unclear how the choice of a patchwise sampling strategy affects the performance of the Alzheimer’s Disease (AD) classification. To this end, the present work investigates the impact of a patchwise sampling strategy for 3D CNN based AD classification. A 3D framework cascaded by two-stage subnetworks was used for AD classification. The patch-level subnetworks learned feature representations from local image patches, and the subject-level subnetwork combined discriminative feature representations from all patch-level subnetworks to generate a classification score at the subject level. Experiments were conducted to determine the effect of patch partitioning methods, the effect of patch size, and interactions between patch size and training set size for AD classification. With the same data size and identical network structure, the 3D CNN model trained with 48 × 48 × 48 cubic image patches showed the best performance in AD classification (ACC = 89.6%). The model trained with hippocampus-centered, region of interest (ROI)-based image patches showed suboptimal performance. If the pathological features are concentrated only in some regions affected by the disease, the empirically predefined ROI patches might be the right choice. The better performance of cubic image patches compared with cuboidal image patches is likely related to the pathological distribution of AD. The image patch size and training sample size together have a complex influence on the performance of the classification. The size of the image patches should be determined based on the size of the training sample to compensate for noisy labels and the problem of the curse of dimensionality. The conclusions of the present study can serve as a reference for the researchers who wish to develop a superior 3D patch-based CNN model with an appropriate patch sampling strategy.

## 1. Introduction

The study of human brain using neuroimaging technologies (generally including magnetic resonance imaging (MRI) and positron emission tomography (PET)) helps the discovery of brain abnormalities in structure and function [1]. Machine learning-based diagnostic image analysis has been widely applied to assist physicians in achieving efficiency and diagnostic accuracy in clinical practice [2,3]. In general, a machine learning algorithm, which typically employs voxel-wise or regional neuroimaging data as the input features, is used to learn the feature patterns of brain diseases [4]. However, the traditional machine-learning method is laborious and relies on well-designed and handcrafted features. By contrast, due to the benefits of huge image datasets and parallel computing technology, deep learning has given rise to remarkable progress in terms of various computer vision tasks [5]. The critical difference between deep learning and traditional machine learning algorithms is that deep learning is capable of automatically detecting and learning complex features and patterns from large datasets. In an era characterized by big neuroimaging data and booming machine learning, deep learning has rapidly emerged as the preferred approach for neuroimage analysis [6,7]. Convolutional neural networks (CNNs) are among the representative networks of deep learning [8,9]. One possible solution for analyzing neuroimaging datasets with small sample sizes is transfer learning, in which two-dimensional (2D) CNN models pretrained from large-scale natural image datasets are applied to solve classification and segmentation tasks [10,11]. However, the 2D approach tends to neglect the three-dimensional (3D) contextual information among the different slices, thereby failing to take full advantage of the neuroimaging information. Because 3D CNNs can capture the 3D structure of a brain image better than 2D CNNs, researchers have turned their attention to 3D CNN models, in an effort to utilize richer spatial 3D information.

To date, the 3D CNNs have achieved certain progress in relation to neuroimage analysis (detection, classification, segmentation, etc.) [12,13,14,15]. Based on the type of input images, the 3D CNNs used for neuroimaging analysis can be divided into two types: image-patch level and subject-level (whole brain images used as inputs). By comparing over 30 studies concerning Alzheimer’s Disease (AD) classification based on CNNs, Wen et al. [16] discovered that different 3D models exhibited similar performances, but the 2D models typically showed poor performance. Although subject-level 3D CNNs are currently favored, millions of voxels are used in the associated computation, which normally requires large training data for efficient training and is considered experimentally impractical. In comparison, image-patch-based methods extract a number of separate image patches from a single 3D image as the network input, each image patch is classified, and finally, the classification results are integrated to obtain the subject-level result. Several studies [17,18] have shown that the classification model based on image patches exhibits better performance than the subject-level approaches in terms of AD classification when the data are limited. In a recent study, Madala and Chandrasekaran [19] found that CNNs do not require full images to be used during training, CNNs trained by using small tiles can match or even surpass the performance of CNNs trained on full images. A 2D theoretical model verified that the curse of dimensionality can be overcome by learning small image tiles. The patchwise sampling strategy for AD classification can be divided into three types: cubic image patches, cuboid image patches, and region of interest (ROI) image patches.

(1) Cubic image patches: Kruthika et al. [20] trained a sparse autoencoder to learn the features of 7 × 7 × 7 image patches, and constructed a 3D CNN model for the AD classification. In another study, Li et al. [21] uniformly divided the brain into 27 local regions, extracted image patches (32 × 32 × 32) from each local region at a stride of 2, grouped the image patches from the same region together by using the K-means clustering method, and then constructed a 3D DenseNet model for learning.

(2) Cuboid image patches: Cheng et al. [17] extracted 27 50 × 41 × 40 local image patches from an MRI image at a stride of 20, constructed a 3D CNN with the same structure to learn and train each image patch, and then applied ensemble learning to achieve the AD classification. In a multimodality study [22], a cascaded CNN was used to learn the multilevel and multimodal features of MRI and PET. Each image was divided into 3 × 3 × 3 parts of 27 overlapping patches (50 × 41 × 40). Multiple VGG-like 3DCNNs were constructed to extract compact, high-level features from each patch. After that, a 2D-CNN was cascaded to ensemble the high-level features of the corresponding image patches from the multimodality images. Zhang et al. [23] divided the brain into 96 image patches (96 × 120 × 96). Then, 3D DenseNet with a connection-wise attention mechanism was applied to extract high-level features from image patches.

(3) ROI image patches: In general, the selected regions of interest (ROIs) are those brain regions most highly correlated with the disease’s development. For instance, the hippocampus region is most commonly selected as an ROI in AD studies [24,25]. By using the hippocampus as an ROI image patch, Liu et al. [26] combined a 3D UNet and a 3D DenseNet to learn the features and realized both the hippocampus segmentation and the disease classification. Huang et al. [27] proposed two 3D VGG-like CNNs to integrate the hippocampus features from both MRI and PET.

Previous studies have applied the image-patch method when investigating various diseases of the nervous system. However, the way in which the choice of patchwise sampling strategy affects 3D CNN-based classification performance remains unknown. In this study, we attempted to investigate the effect of patch sampling strategy on classification performance by classifying AD and cognitively normal (CN) brain images.

## 2. Materials and Methods

### 2.1. Data-Set

Data employed in the preparation of this article were obtained from the Alzheimer’s Disease Neuroimaging Initiative (ADNI) database (http://adni.loni.usc.edu/) accessed on 1 January 2022. The ADNI was launched in 2003 as a public-private partnership, led by Principal Investigator Michael W. Weiner, MD. The primary goal of ADNI has been to test whether MRI, PET, other biological markers, and clinical and neuropsychological assessment can be combined to measure the progression of mild cognitive impairment and AD.

High-resolution brain structural MRI (sMRI) scans were collected at multiple ADNI sites using a 1.5-T system from GE Healthcare, Philips Medical Systems, or Siemens Healthcare, depending on the scanning site. T1-weighted volumetric MP-RAGE data were collected for each subject, and the raw DICOM images were downloaded from the public ADNI site (http://www.loni.ucla.edu/ADNI/Data/index.shtml (accessed on 1 January 2022)). Parameter values vary by study scanning site and can be accessed at http://www.loni.ucla.edu/ADNI/Research/Cores/ (accessed on 1 January 2022). Only baseline scans were used for the study. The population in this study included ADNI-1 participants enrolled in the CN or AD cohorts, including 187 AD patients and 229 CNs at baseline. Demographic details of the two groups are provided in Table 1, including age, gender, years of education, and mini-mental state examination (MMSE).

### 2.2. Image Preprocessing

**Figure 1:**
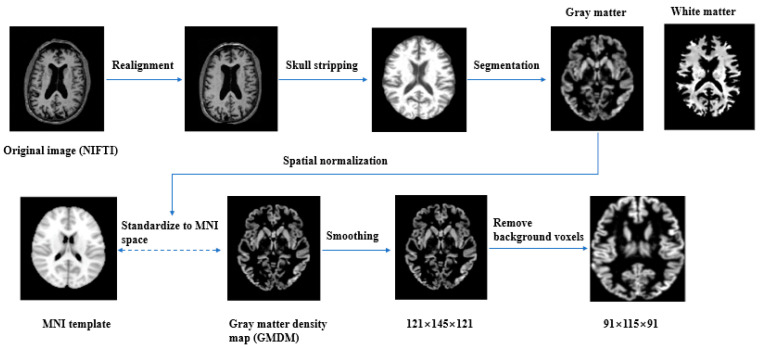
Preprocessing pipeline.

The downloaded data were first converted from DICOM to Neuroimaging Informatics Technology Initiative (NIFTI) format, by using MRIcron software (http://people.cas.sc.edu/rorden/mricron/index.html (accessed on 1 January 2022)). Images were manually reoriented to place their native-space origin at the anterior commissure. Images were then preprocessed by using the Computational Anatomy Toolbox (CAT12) toolbox (http://www.neuro.uni-jena.de/cat/ (accessed on 1 January 2022)), an extended toolbox of SPM12 [27] with default settings. The preprocessing pipeline included realignment, skull stripping, segmentation into gray matter and white matter, and finally, the segmented gray matter images were spatially normalized into the Montreal Neurological Institute (MNI) space by using diffeomorphic anatomical registration by using exponentiated Lie algebra nonlinear normalization and modulated to preserve volume information. The modulated and warped 3D gray matter density maps (GMDMs) were smoothed by using a 3D Gaussian kernel of 2 mm full width at half maximum. The GMDMs had a dimensionality of 121 × 145 × 121 in the voxel space, with the voxel size of 1.5 × 1.5 × 1.5 mm^3^. The background voxels increased the computational complexity of model, but they did not contribute to the classification performance. Thus, we established a new bounding box with the dimension of 91 × 115 × 91 (voxel size of 1.5 × 1.5 × 1.5 mm^3^), which removed most of the background voxels. The complete preprocessing pipeline is summarized in Figure 1.

### 2.3. Patch Extraction

**Figure 2:**
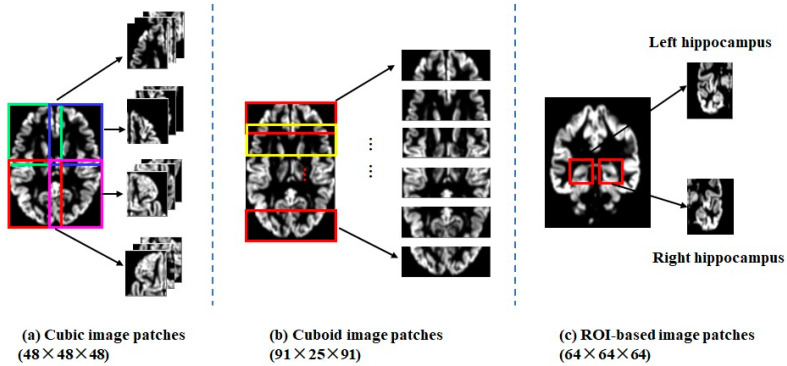
Three partition methods for patch extraction (Note: bounding boxes with different colors represented image patches taken from different positions). (**a**). cubic image patches; (**b**). cuboid image patches; (**c**) ROI-based image patches.

The patchwise sampling strategy involved the following three partition methods for the whole brain images.

(1). Cubic image patches: Twelve 48 × 48 × 48 local image patches, which were partially overlapped, were sampled to cover the whole brain, as shown in Figure 2a.
(2). Cuboid image patches: Six 91 × 25 × 91 local image patches, which were also partially overlapped, were sampled along the coronal axis, as shown in Figure 2b.
(3). ROI patches: Two 64 × 64 × 64 image patches were sampled to cover the left (or right) hippocampus with certain margins, as shown in Figure 2c.

In addition, the whole brain image was sectioned into cubic patches of different sizes (8 64 × 64 × 64, 28 32 × 32 × 32 and 72 24 × 24 × 24 image patches) from the GMDMs, respectively, in an effort to determine how the patch size influenced the model’s performance.

### 2.4. Network Architecture

#### 2.4.1. Subject-Level CNNs

Subject-level CNNs (Table 2) had VGGNet-like 3D CNN structures. The CNN comprised four convolutional layers with channels of 8, 16, 32, and 64 channels. All convolutional layers had a kernel of 3 × 3 × 3, and a unit stride with zero-padding, followed by L2 regularization and rectified linear unit (ReLU) activation. Each Conv layer was followed by a max-pooling layer. The first max-pooling layer had a size of 3 × 3 × 3, and the stride was set to 3. In addition, the other three max-pooling layers had a size of 2 × 2 × 2 with a stride of 2. As the tail of the CNN model, the number of neurons in three fully connected (FC) layers were 1024, 128, and 2, respectively. The last FC layer determines the final probability score with a Softmax activation. We applied dropout to the first two FC layers to avoid over-fitting. To conveniently compare the performance of the tested patch-level approaches with the subject-level approach, the 3D subject-level CNN was treated as the baseline model. This baseline model was trained by using a grid-search technique in order to find the optimal combination of hyperparameters (learning rate, batch size, dropout ratio, number of epochs) for this architecture. The range of the hyperparameter values was (1 × 10^−8^–1 × 10^−2^ for learning rate, 12–48 for batch size, 0.3–0.8 for dropout ratio, and 100–1000 for the number of epochs). In the grid search, fivefold cross-validation (CV) was performed on the training set. While changing the values of the hyperparameter, mean values for accuracy (ACC) were calculated for each value of the hyperparameter. The value of the hyperparameter that maximized the mean ACC value was used. The loss function was binary cross-entropy. The learned hyperparameters are shown below. The Adam optimizer had a learning rate of 1 × 10^−4^. The batch size was 24, dropout ratio was 0.5, and the number of epochs was set to 300.

#### 2.4.2. Image Patch-Level CNNs

**Figure 3:**
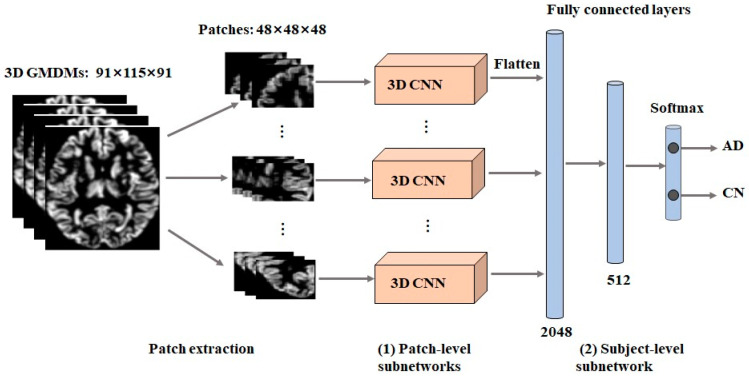
An overview of the image patch-level 3D CNN framework, which includes two components: patch-level subnetworks, and a subject-level subnetwork.

The image patch-level classification framework (Figure 3) adopted a set of local image patches as the inputs. Moreover, it was based on a cascaded CNN consisting of two components: patch-level subnetworks and a subject-level subnetwork. Briefly put, the patch-level subnetworks were used to generate feature representations and classification scores for these image patches. Then, all the learned feature representations were integrated and processed by the subject-level subnetwork, which allowed the classification result to be obtained.

- Patch-level subnetworks

For the patch-level subnetworks, the model architecture was basically identical to the baseline model, although the convolution kernel of the first max-pooling layer was altered to 2 × 2 × 2, and the stride was set to 2. The initial learning rate, batch size, and dropout ratio of patch-level networks were kept the same as in the baseline model. The epoch number is set to 200, because patch-level networks have a faster convergence speed.

- Subject-level subnetwork

The convolutional layers in patch-level subnetworks worked as local feature extractors that combined low-level features into high-level features. The subject-level subnetwork comprised three FC layers, which were utilized for integrating 3D information; dropout was included to prevent the overfitting of the training model, and the dropout ratio was set at 0.5. Deep features from Conv4 learned by the patch-level subnetworks were concatenated and fed into the three FC layers for subject-level classification. The number of neurons in the three FC layers was 2048, 512, and 2, respectively. ReLU activation and L2 regularization were added to FC1 and FC2. The last FC layer used SoftMax activation to generate a subject-level classification score.

### 2.5. Experiments and Implementation

The data utilized in the experiment were randomly divided into the training set (70%), validation (10%), and testing sets (20%) and adopted an undersampling technique was adopted to overcome an imbalance problem present in the testing set. During subdivision, the class distributions of those datasets were kept the same as those of the original class distribution. The mean voxel-wise absolute intensity differences between all GMDMs of the CN class in the testing set and the GMDMs of the AD class in the testing set were computed. For each example in the AD class, one instance from the CN class was selected that had minimal mean voxel-wise absolute intensity differences from it, and undersampling was implemented until the balance of class distribution in the testing set was achieved. The process of random division and undersampling was repeated 20 times. The experimental results were averaged over 20 tests.

All classification models used Python 3.7 as the programming language, Tensorflow 2.0 as the deep learning algorithm programming framework, and the models’ training and testing were performed on a workstation equipped with an NVIDIA GeForce GTX 1080 GPU in a Windows environment.

Each patch-level subnetwork was trained separately, and the network weight was randomly initialized. In the training process of the subject-level subnetwork, we locked all the convolutional layers of the pretrained patch-level subnetworks. In the cost function calculation, balanced class weights were used to ensure that classes were weighted inversely proportional to their frequency in the training set. We adopt an early stop strategy that stops training when the validation metric does not show improvement for 20 consecutive epochs.

Five evaluation indexes, ACC, sensitivity (SEN), specificity (SPE), F1-score, and AUC were used in this study. TP, TN, FP, and FN denoted the quantity of true positive, true negative, false positive, and false negative, respectively. The calculation formulas are as follows:

(1) $$\mathrm{ACC}=\frac{\mathrm{TP}+\mathrm{TN}}{\mathrm{TP}+\mathrm{TN}+\mathrm{FN}+\mathrm{FP}}\;\;$$

(2) $$\mathrm{SEN}=\frac{\mathrm{TP}}{\mathrm{TP}+\mathrm{FN}}\;\;$$

(3) $$\mathrm{SPE}=\frac{\mathrm{TN}}{\mathrm{TN}+\mathrm{FP}}\;$$

(4) $$F1-\mathrm{score}=\frac{2\times \mathrm{TP}}{2\times \mathrm{TP}+\mathrm{FP}+\mathrm{FN}}\;.$$

To check the statistical differences among the ACC for all the models, we computed one-way repeated-measures ANOVA with Tukey post hoc analysis for multiple comparisons. Greenhouse–Geisser sphericity correction was made if Mauchly’s test of sphericity indicated a violation of the sphericity assumption for the repeated-measures ANOVA tests.

## 3. Results

### 3.1. The Influence of Partition Methods

Classification performance was compared between the CNNs trained with the three partition methods and the baseline model in the AD vs. CN classification task (Table 3). All four classifiers performed well, with ACC exceeding 85%. The one-way repeated-measures ANOVA indicated that ACC was significantly affected by the partition methods (one-way ANOVA: F = 8.247, *p* < 0.001). The model trained with cubic (48 × 48 × 48) image patches showed the best performance in all five evaluation indices, with an ACC of 89.6%, indicating a 2.2% improvement over the baseline model. A post hoc test revealed that the model with 48 × 48 × 48 image patches had a statistically higher ACC than the other models (*p* < 0.01). Although a 91 × 25 × 91 image patch occupies a similar volume to a 48 × 48 × 48 image patch, the model trained with this patch size did not achieve the same performance (ACC = 86.8%). For ROI-based image patches extracted with the hippocampus as the central region, the performance (ACC = 87.6%) achieved was comparable to whole-brain image patches by using only two 64 × 64 × 64 patches (left hippocampus and right hippocampus).

### 3.2. The Influence of Image Patch Size

The size of the patches plays an important role in the patchwise sampling strategy. We started the experiments with patches of size 24 × 24 × 24, and found that gradually increasing the size of the patches up to 64 × 64 × 64 resulted in improved performance (Table 4). The 48 × 48 × 48 image patches achieved the best performance (ACC = 89.6%). The 24 × 24 × 24 image patches were comparable to the 32 × 32 × 32 image patches in terms of ACC. There were significant differences in classification performance among algorithms based on ACC (one-way ANOVA: F = 4.447, *p* < 0.01). A post hoc test showed that the model with 48 × 48 × 48 image patches had a statistically higher ACC than the models with 24 × 24 × 24 and 32 × 32 × 32 image patches (*p* < 0.05).

### 3.3. The Relationship between Image Patch Size and Training Sample Size

The image patch size and the training sample size show a complex relationship with model performance. When the training set was reduced by half, the model became more susceptible to overfitting. All patch-level models and the baseline model experienced performance degradation (Table 5). The performance degradation was smallest for the 24 × 24 × 24 image patches, which maintained an ACC of 87.1%, and largest for the 48 × 48 × 48 image patches, which achieved 4.1%, when comparing the performances for the complete training set and half of the training set. The AUC of the models with 32 × 32 × 32 and 64 × 64 × 64 image patches and the baseline model decreased by about 3%. After halving the training sample, there was no significant difference in ACC t between the models (one-way ANOVA: F = 1.825, *p* = 0.136).

## 4. Discussion

In general, if the whole brain’s information is used as the input with a sufficient training sample size, the 3D network with the greatest depth and width will exhibit superior performance [28]. In neuroimaging-based studies, there are normally a limited (e.g., hundreds) number of subjects with a very high number (millions) of dimensional features, which greatly increases the risk of overfitting. It is anticipated that an optimized patch-wise sampling strategy may provide a clue for improving the AD classification model, this, however, has not been verified. In this study, we investigated the effect of a patch-wise sampling strategy on the performance of a 3D CNN model for AD classification.

### 4.1. ROI Patches

The empirically defined brain regions have the features with greater discriminability. For example, the temporal lobe cortex, the amygdaloid nucleus, and the hippocampus are the most severely affected regions in AD. Among them, hippocampus atrophy is implicated in both memory and learning. In this study, the diagnostic model that relied on predetermined hippocampal ROIs demonstrated a suboptimal effect when compared with the other models. The interpretation of the results is simple and intuitive. First, although the hippocampus is the most important region in terms of AD, hippocampus ROI cannot cover all possible pathological features of the whole brain. Some potentially important brain regions affected by AD could be ignored. Secondly, diagnostic models based on empirically predetermined ROIs are influenced by the heterogeneity of the disease. AD is a heterogeneous disease [29], the hippocampal area is well preserved in some AD patients [30]. For those patients, the hippocampus ROIs do not represent a good choice for image patches. 

### 4.2. The Effect of Patch Shape

When compared with ROI patches, patches generated from random partitioning attempt to learn local-to-global feature representations for whole brain MRI. Although cubic image patches and cuboid image patches basically contain the same quantity of voxels, their classification capacities are quite different. More specifically, cubic image patches exhibit better classification performance. The influence of a patchwise sampling strategy on the classification performance may be due to the regional distribution of the pathology in AD. In a study for AD diagnosis, Liu et al. [31] adopted a data-driven learning approach to discover disease-related anatomical landmarks. They found that many landmarks with high discriminative capability were close to each other, and those landmarks were more concentrated in certain AD related brain regions. When the volume of an image patch is fixed, if its shape has the form of a cube, the average Euclidean distance between any two points in the image patch is the shortest. For example, over 10 million Monte Carlo runs, we found that the average distance between any two points in a 48 × 48 × 48 cubic was 47.63 mm, and the average distance between two points in a 91 × 25 × 91 cuboid was approximately 73.25 mm. That means when the volume of patches is the same, the label of the cuboid cubic division might be more noisy compared with the cubic division. However, it must be acknowledged that the better performance observed in this study on the part of cubic patches might not be maintained in relation to other brain diseases.

### 4.3. The Relationship between Image Patch Size and Training Sample Size

Three-dimensional CNNs have the potential to retrace the success story of 2D-CNNs, but they have two major drawbacks in terms both of high computational cost and the curse of dimensionality. More data are required to create a model that meets the problem requirements and criteria as the problem dimensionality increases. For most of the neuroimaging studies, it is very hard to acquire a large neuorimage dataset. An option to decrease the effect of the curse of dimensionality is to use small image patches. When an image patch is too small, the labels are noisy. When an image patch is too large in size and the number of training samples is insufficient, CNN easily suffers from the curse of dimensionality problem. In terms of the input patches, several different sizes were tested in this study. Unsurprisingly, the patches with a medium size (48 × 48 × 48) were able to achieve the best performance for the whole training set. Furthermore, the performance of the model was also evaluated when the training data were reduced by half. It was determined that the model’s performance was degraded no matter what size of image patch was used as the input. The performance degradation amplitude in the case of the smallest image patches (24 × 24 × 24) was the minimum. The image patch size should be reduced to mitigate the curse of dimensionality problem. 

### 4.4. Performance Comparison

Although the goal of this study is not to develop a superior model, it is intended to provide guidance to researchers who wish to develop a superior 3D patch-based CNN model with an appropriate patch sampling strategy. Table 6 shows the comparison between this study and state-of-the-art approaches. The model is comparable to a state-of-the-art approach, especially considering that we used a more rigorous experimental design, employed a downsampling strategy to balance classes in the test set, and selected only CN subjects that are difficult to distinguish from AD. 

### 4.5. Limitations

It must be acknowledged that the present study has a number of limitations. First, some image patches have limited discriminative power, which means that selecting appropriate image patches may boost the performance of the model. Therefore, some of the conclusions drawn in this study may need to be modified by a network pruning strategy. Secondly, the batch size controls the number of samples propagating through CNNs during training. In the current implementation, the training batch size is fixed. Tuning the batch size affects the training loss curve and computation efficiency. As image patches get smaller, the batch size during training can be increased, which could make the training more efficient. Thirdly, given that the structural changes caused by AD vary according to the severity of the disease, it would be useful to boost the model’s performance by extending the method to multiscale image patches. Fourth, because many researchers [17,22,27] prefer to choose a relatively old but structurally simple and highly efficient VGG architecture to implement their patch-based 3D CNNs, we investigated VGG-like 3D CNNs in this study. The experimental results can be theoretically supported by the 2D theoretical framework [19], but whether they can be extrapolated to more complex 3D models requires further experimental validation. Fifth, the requirement for more public datasets for AD research is highlighted by the importance of building a robust classification model on new and unseen data.

## 5. Conclusions

By data mining on the AD database by using an optimal CNN model, a valuable computer-aided AD diagnosis system is very promising and feasible for clinic use in the future. In this study, we investigated the effect of a patchwise sampling strategy for 3D CNN based AD classification. When the pathological features are concentrated in specific brain regions, the empirically predetermined ROI patches are optimal, which can be verified by studying other homogeneous diseases in the future. The experimental results show that the cubic image patches perform better than the cuboid image patches in classifying AD, which is probably related to the regional distribution of pathology in AD. In this study, the 48 × 48 × 48 image patches performed best for the entire training set, and the 24 × 24 × 24 image patches performed best for one half of the training set. The size of the image patch should be determined based on the size of the training sample to compensate for noisy labels and the curse of dimensionality.

## Figures and Tables

**Table 1:** Demographic information of the subjects in ADNI-1.

Characteristic	AD	CN
Subjects	187	229
Age	75.26 ± 7.53	75.87 ± 5.02
Gender (Male/Female)	98/89	119/110
Education	14.66 ± 3.14	16.07 ± 2.85
MMSE	23.28 ± 2.04	29.11 ± 1.00

The age, education years, and MMSE values are reported as mean ± standard deviation (Std).

**Table 2:** CNN architecture of subject-level CNN (baseline model).

Layer	Kernel Size	Stride	Output Size	Parameters
Input	-	-	91 × 115 × 91	-
Conv1	3 × 3 × 3	1	31 × 39 × 31	224
Conv2	3 × 3 × 3	1	16 × 20 × 16	3472
Conv3	3 × 3 × 3	1	8 × 10 × 8	13,856
Conv4	3 × 3 × 3	1	4 × 5 × 4	55,360
FC1	1024	-	1 × 1024	5,243,904
FC2	128	-	1 × 128	131,200
FC3	2	-	1 × 2	258

**Table 3:** Results of models based on different image patch partition methods in AD vs. CN classification (mean ± standard deviation).

Patch Size/Partition Method	ACC (%)	SEN (%)	SPE (%)	F1-Score (%)	AUC (%)
48 × 48 × 48/cubic patches	89.6 ± 1.8	89.8 ± 3.6	90.1 ± 4.4	89.6 ± 2.0	89.8 ± 1.9
64 × 64 × 64/ROIs patches	87.6 ± 2.3	86.3 ± 3.2	89.7 ± 5.0	87.8 ± 2.4	87.6 ± 2.2
91 × 25 × 91/cuboid patches	86.8 ± 2.5	85.6 ± 3.2	88.7 ± 4.9	87.0 ± 2.7	86.7 ± 2.4
91 × 115 × 91/baseline	87.7 ± 2.8	87.5 ± 3.7	88.4 ± 4.4	87.7 ± 2.9	87.7 ± 2.7

**Table 4:** Results of models based on different cubic patch sizes in AD vs. CN classification (mean ± standard deviation).

Patch Size	ACC (%)	SEN (%)	SPE (%)	F1-Score (%)	AUC (%)
24 × 24 × 24	87.6 ± 2.2	87.4 ± 4.8	88.8 ± 5.1	87.7 ± 2.4	87.5 ± 2.2
32 × 32 × 32	87.8 ± 2.3	87.5 ± 3.3	88.5 ± 4.2	87.8 ± 2.5	87.8 ± 2.3
48 × 48 × 48	89.6 ± 1.8	89.8 ± 3.6	90.1 ± 4.4	89.6 ± 2.0	89.8 ± 1.9
64 × 64 × 64	87.9 ± 2.0	87.1 ± 3.7	89.3 ± 4.1	88.1 ± 1.9	87.7 ± 2.1

**Table 5:** Results of models based on different image sizes in AD vs. CN classification after halving the training sample size (mean ± standard deviation).

Patch Size/Partition Method	ACC (%)	SEN (%)	SPE (%)	F1-Score (%)	AUC (%)
24 × 24 × 24/cubic patches	87.1 ± 3.1	86.8 ± 5.0	88.1 ± 5.1	87.2 ± 3.6	87.2 ± 3.1
32 × 32 × 32/cubic patches	85.2 ± 4.5	84.4 ± 4.7	86.3 ± 5.2	85.4 ± 4.5	85.1 ± 4.6
48 × 48 × 48/cubic patches	85.5 ± 4.5	85.1 ± 5.5	86.6 ± 5.3	85.6 ± 4.4	85.5 ± 4.7
64 × 64 × 64/cubic patches	84.3 ± 5.5	83.7 ± 5.8	85.6 ± 6.9	84.4 ± 5.7	84.3 ± 5.5
64 × 64 × 64/ROIs	83.6 ± 5.2	83.1 ± 6.0	84.7 ± 6.2	83.7 ± 5.3	83.4 ± 5.3
91 × 115 × 91/baseline	85.0 ± 4.0	84.6 ± 4.6	85.7 ± 5.0	85.1 ± 4.1	84.9 ± 4.2

**Table 6:** Results of state-of-the-art approaches using the 3D patch-level CNN for AD diagnosis.

Articles	Model	Patch Size	Sample Size	Image Modality	ACC (100%)
Chen et al. [17]	3D VGG	50 × 41 × 40	AD: 229, CN: 199	sMRI	87.15
Huang et al. [25]	3D VGG	96 × 96 × 48	AD:647, CN:731	sMRI/FDG-PET	90.10
Kruthika et al. [20]	3D SAE	7 × 7 × 7	AD: 75, CN: 75	sMRI	97.60
Li et al. [21]	3D DenseNet	32 × 32 × 32	AD:199, CN:229	sMRI	89.5
Liu et al. [22]	3D VGG	50 × 41 × 40	AD: 93, CN: 100	sMRI/FDG-PET	93.26
Liu et al. [26]	3D U-Net+3D DenseNet	62 × 48 × 58	AD: 97, CN: 119	sMRI	88.90
Zhang et al. [23]	3D DenseNet with attention	96 × 120 × 96	AD: 280, CN: 275	sMRI	97.35
Proposed approach	3D VGG	48 × 48 × 48	AD: 187, CN: 229	sMRI	89.6

## Data Availability

The dataset is owned by a third-party organization; the Alzheimer’s Disease Neuroimaging Initiative (ADNI). Data are publicly and freely available from the http://adni.loni.usc.edu/data-samples/access-data/ accessed on 1 January 2022. Institutional Data Access/Ethics Committee (contact via http://adni.loni.usc.edu/data-samples/access-data/ (accessed on 1 January 2022)) upon sending a request that includes the proposed analysis and the named lead.
